# Nematode endoparasites do not codiversify with their stick insect hosts

**DOI:** 10.1002/ece3.2264

**Published:** 2016-07-10

**Authors:** Chloé Larose, Tanja Schwander

**Affiliations:** ^1^ Department of Ecology and EvolutionUniversity of LausanneLausanneSwitzerland

**Keywords:** Codiversification, cophylogeny, endoparasite, host–parasite interaction

## Abstract

Host–parasite coevolution stems from reciprocal selection on host resistance and parasite infectivity, and can generate some of the strongest selective pressures known in nature. It is widely seen as a major driver of diversification, the most extreme case being parallel speciation in hosts and their associated parasites. Here, we report on endoparasitic nematodes, most likely members of the mermithid family, infecting different *Timema* stick insect species throughout California. The nematodes develop in the hemolymph of their insect host and kill it upon emergence, completely impeding host reproduction. Given the direct exposure of the endoparasites to the host's immune system in the hemolymph, and the consequences of infection on host fitness, we predicted that divergence among hosts may drive parallel divergence in the endoparasites. Our phylogenetic analyses suggested the presence of two differentiated endoparasite lineages. However, independently of whether the two lineages were considered separately or jointly, we found a complete lack of codivergence between the endoparasitic nematodes and their hosts in spite of extensive genetic variation among hosts and among parasites. Instead, there was strong isolation by distance among the endoparasitic nematodes, indicating that geography plays a more important role than host‐related adaptations in driving parasite diversification in this system. The accumulating evidence for lack of codiversification between parasites and their hosts at macroevolutionary scales contrasts with the overwhelming evidence for coevolution within populations, and calls for studies linking micro‐ versus macroevolutionary dynamics in host–parasite interactions.

## Introduction

Parasites are ubiquitous in nature and are known to play a fundamental role in community ecology and the evolution of the hosts they infect (e.g., Thompson 1994; Bohannan and Lenski 2000; Woolhouse et al. 2002; Decaestecker et al. 2005; Schmid‐Hempel 2011). By definition, parasites have a negative effect on host fitness, favoring selection of enhanced defense or resistance mechanisms in the hosts. In turn, host defense mechanisms are generally detrimental for parasites, leading to selection for counteradaptations in the parasites. Host–parasite coevolution thus stems from reciprocal selection on host resistance and parasite infectivity (e.g., Thompson 1994; Ebert 1998; Clayton et al. 1999; Carius et al. 2001; Dybdahl et al. 2014). Evidence that coevolutionary interactions drive evolutionary changes stems from taxonomically diverse host systems, including bacteria (e.g., Weitz et al. 2005), plants (e.g., Dodds and Rathjen 2010; Karasov et al. 2014), invertebrates (e.g., Decaestecker et al. 2007; Ebert 2008), and vertebrates (Kerr 2012). As a consequence, host–parasite coevolution is widely seen as a major driver of diversification, the most extreme case being codiversification or parallel speciation in hosts and their associated parasites (e.g., Clarke 1976; Price 1980; Kiester et al. 1984; Buckling and Rainey 2002; Thompson et al. 2005; Nieberding and Morand 2006; Ricklefs 2010; Yoder and Nuismer 2010; Weber and Agrawal 2012; Masri et al. 2015).

Codiversification is particularly expected for endoparasites (more than for ectoparasites) given their direct interaction with the host immune system (Poinar 1974; Poulin 2007; Cressler et al. 2014, 2015). Here, we report on endoparasitic nematodes which infect different species of stick insects in the genus *Timema*. *Timema* are herbivorous, wingless stick insects native to the western part of the United States (Vickery 1993). We discovered endoparasitic nematodes serendipitously when collecting *Timema* stick insects in the field; an individual nematode larva occasionally emerged from a *Timema* host, killing its host in the process. This parasitic infection thus induces a dramatic cost on host fitness. We presumed that these nematodes belong to the Mermithidae family, given their ecology (Poinar et al. 1976) and morphology (Poinar 1975; Presswell et al. 2015). Mermithid nematodes are mainly known as endoparasites of insects (Kaiser 1991; Nikdel et al. 2011), and occasionally of other invertebrates (Vandergast and Roderick 2003). Their life cycles vary among species, but females of terrestrial species typically lay eggs in the soil during periods of high moisture. Preparasites (corresponding to larval stage four) then hatch from eggs and migrate to the surface in search of a suitable host. When a preparasite finds a host, it enters the host's hemocoel through a hole pierced into the cuticle and develops in the hemocoel while feeding from the hemolymph (Poinar et al. 1976; Colbo 1990). The fully developed mermithid larvae then emerge through the intersegmental joints of the host, killing the host in the process. After emergence, the free‐living, nonfeeding postparasites burrow in the soil where they molt to the adult stage, mate, and lay eggs (Poinar and Otieno 1974).

We found mermithid‐like endoparasitic nematodes in nine different *Timema* stick insect species, which prompted us to test for codiversification of these nematodes and their hosts. We infer the most probable evolutionary events that have generated the present distribution of parasite lineages among the different host species. This allows us to test whether adaptation to different host species has contributed to endoparasite diversification.

## Materials and Methods

### Sample collection and molecular methods

**Figure 1:**
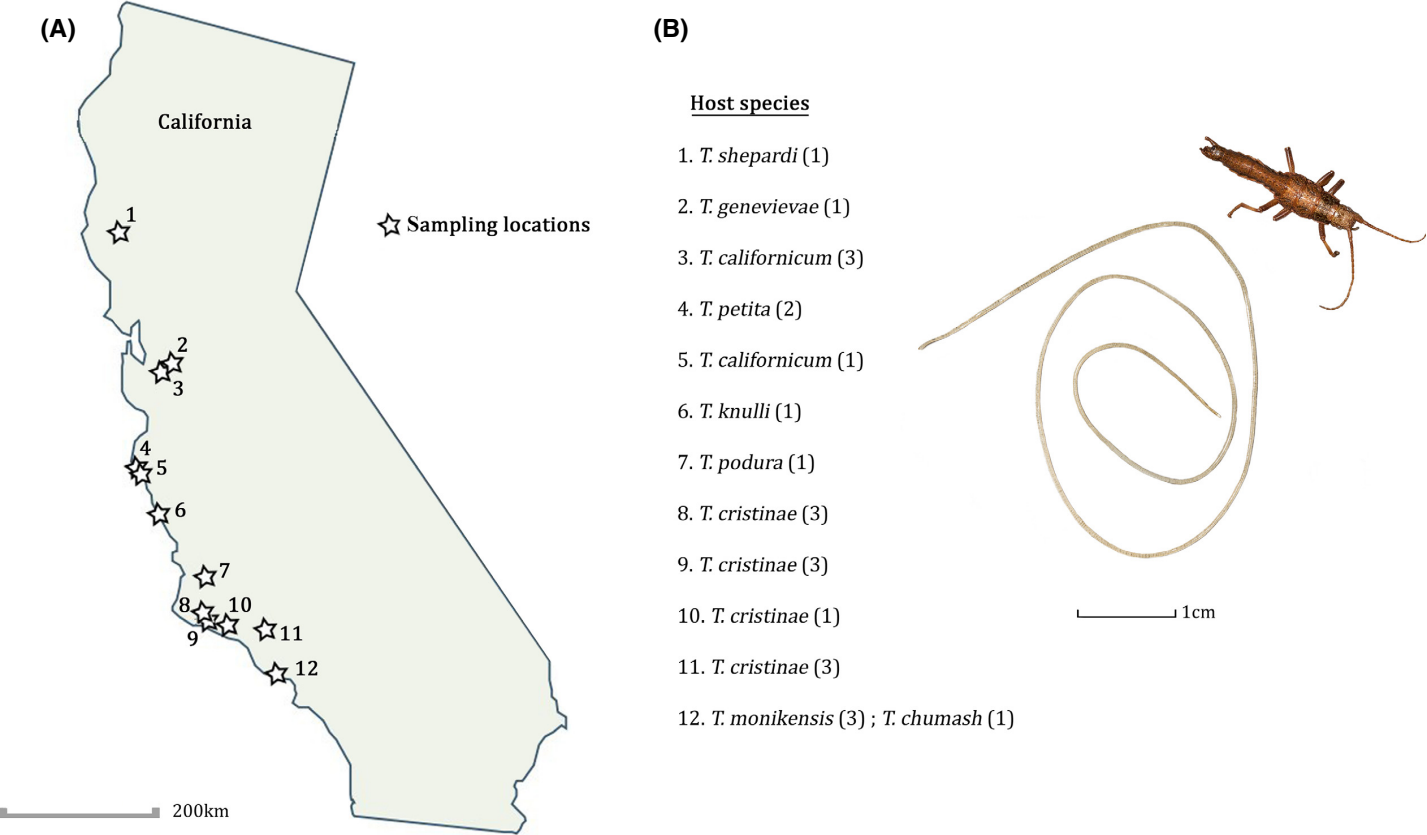
(A) Locations of the endoparasitic nematodes sampled in this study. Numbers in brackets indicate the number of nematodes per host species and location. Please note that the large number of nematodes collected from the *T. cristinae* host is explained by *T. cristinae* being the most intensively sampled host species (not by this species being more infected than others). (B) Picture of an endoparasitic nematode after it exited and killed its *Timema* host.

*Timema* stick insects from 13 different species (Fig. 1A) were collected throughout California, between 2007 and 2015, in order to perform a number of experiments not related to the present study. While maintaining stick insects in the laboratory, we occasionally found parasitic nematodes that emerged from an individual female stick insect, killing its host in the process. Females from which the nematodes emerged died before producing a single egg and had undeveloped ovaries, indicating that these nematodes completely impede reproduction of their host. Each emerged nematode was collected and stored in 95% alcohol until further use. Even though thousands of stick insects were collected over the 9 years, we only assembled a set of 31 nematodes from nine of the 13 sampled *Timema* stick insect species, with a nematode emerging from 0 to 1.2% of host individuals, depending on years and host species. These emergence rates only consider nematodes that successfully developed within their hosts and do not take into account cases where nematode development would have been suppressed by the host's immune system. Furthermore, given the size of the nematodes (Fig. 1B), undetected emergences among the collected stick insects are highly unlikely, an assumption confirmed by the dissection of 821 stick insects of which fewer than 1% were infected (2 out of 821). We therefore tested for host–parasite coevolution between the endoparasitic nematodes and their *Timema* hosts with multiple nematodes available for four host species. For one of these, the most intensively sampled host (*T. cristinae*), we had 16 nematodes, of which we used nine for our study (three from each of three different host populations), for a total of 24 nematodes from nine different *Timema* species (Fig. 1A).

DNA from the nematodes was extracted using a QIAGEN AG (Hombrechtikon, Switzerland) DNeasy Blood & Tissue kit following the manufacturer's protocol. We used two primer pairs from other studies for amplifying a ~1200‐bp portion of the 18S small ribosomal subunit (18S rRNA): the universal SSU primers SSU18A (5’‐AAAGATTAAGCCATGCATG) and SSU26R (5’‐CATTCTTGGCAAATGCTTTCG) from Blaxter et al. (1998) and 18S‐5F (5’‐GCGAAAGCATTTGCCAAGAA) and 18S‐9R (5’‐GATCCTTCCGCAGGT TCACCT) from Vandergast and Roderick (2003). PCRs were performed in 25**** *μ*L containing 0.5 U AmpliTaq DNA Polymerase (Applied Biosystems, Foster City, CA), 1.8 mmol/L MgCl2, 0.2 mmol/L each dNTP, and 0.4 mmol/L each primer. For both primer pairs, a touchdown PCR protocol was employed. The first 10 cycles were performed with denaturation at 95°C for 30 sec, annealing at 55°C for 30 sec, and an extension of 40 sec at 72°C. Ten additional cycles were run with an annealing temperature of 50°C and the 20 final cycles with an annealing temperature of 45°C. Ten‐min final extension at 72°C ended the amplification. PCR products were visualized on agarose gels stained with ethidium bromide. Five *μ*L of each PCR product were purified using 4 *μ*L of ExoI (20 U/*μ*L) (Thermo Scientific, Life Technologies Europe B.V., Nieuwerkerk aan den IJssel, Zweigniederlassung Zug, Switzerland) mixed with FastAP Thermosensitive Alkaline Phosphatase (1 U/*μ*L) (Thermo Scientific). After addition of 5 *μ*L (5 mmol/L) forward primer, purified PCR products were sent to *GATC Biotech, Germany* (www.gatc-biotech.com) for Sanger sequencing. We aligned the 18S rRNA portions using the algorithm MUSCLE (Edgar 2004) as implemented in SeaView 4.5.4 (Galtier et al. 1996; Gouy et al. 2010). The final alignment consisted of 1078 bp (including 7–26 bp gaps). GenBank accession numbers are indicated in Table S1.

### Phylogenetic placement of the endoparasitic nematodes

**Figure 2:**
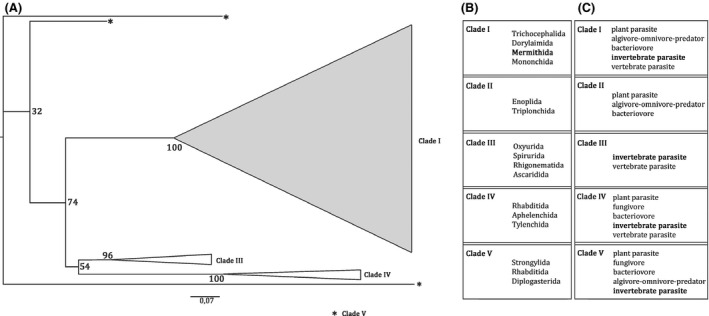
Phylogenetic placement of endoparasitic nematodes from *Timema* within the Nematoda phylum. (A) Maximum‐likelihood phylogeny based on the 18S rRNA sequence of 57 nematodes. The highlighted group corresponds to Clade I, which comprises the 24 *Timema* endoparasitic nematode sequences (see Fig. 3 for details of this clade). Numbers associated with branches indicate bootstrap support (1000 replicates). (B) Nematode orders described in each clade and (C) their trophic ecologies. Information indicated in (B) and (C) are from Blaxter et al. (1998).

To verify that the *Timema* endoparasitic nematodes indeed belong to the Mermithidae family, we built a maximum‐likelihood phylogeny using the newly generated 18S rRNA sequences and published sequences from Ross et al. (2010). The published sequences were chosen to represent the four nematode clades proposed by Blaxter et al. (1998), which are known to comprise endoparasitic nematodes (“Clades I, III, IV, and V”; see Fig. 2C). For the first clade (“Clade I” in Blaxter et al. 1998), which includes the Mermithidae family (Fig. 2B), we used 24 sequences. Three representative sequences per clade were used from the three remaining clades (“Clade III” to “Clade V” in Blaxter et al. 1998), for a total of 33 sequences. Details for each sequence, including GenBank accession numbers, are shown in Table S1. Using likelihood scores as implemented in FindModel (Posada and Crandall 1998; Tao et al. 2005), we inferred that the GTR+G model best described our dataset (LnL = −6947, AIC = 13912). We used this model to construct a maximum‐likelihood tree in SeaView 4.5.4 (Galtier et al. 1996; Gouy et al. 2010) with heuristic searches (excluding gaps). The bootstrap support for each branch was calculated using the same model with 1000 replicates.

We also tested whether *Timema* endoparasites are closely related to the *Clitarchus* stick insect endoparasite found by Yeates and Buckley (2009) by adding the 18S sequence portion from that species to the sequence set described above and running the same phylogenetic analyses. However, because the *Clitarchus* 18S sequence portion was much smaller (781 bp) than the amplified portion in *Timema* (1200 bp) and thus less informative, we did not use this sequence for any further analyses.

### Host–parasite cophylogenetic analyses

We used two cophylogeny methods to infer the most probable coevolutionary history between *Timema* and their endoparasitic nematodes: the method implemented in the program TreeMap 3.0*β* (Page 1994; Charleston 1998; Charleston and Page 2002), and the one implemented in Jane 4.0 (Conow et al. 2010). Both methods reconcile tree topologies of hosts and parasites by inferring four or five (depending on the method) evolutionary events: (1) “codivergence,” which occurs when the host and parasite diverge simultaneously; (2) “duplication,” which corresponds to the divergence of the parasite, with both descendants of the parasite lineage remaining associated with the same host; (3) “host switch,” which is a duplication followed by the shift of one parasite lineage to a new host; (4) “parasite loss,” which corresponds to the apparent absence of a parasite lineage in the descendants of a host that previously had an associated parasite; and (5) “failure to diverge,” which occurs when a host speciates but the parasite does not (the same parasite remains on both new host species). Each of these evolutionary events is given a cost related to the likelihood of that event (Ronquist 1997), and cophylogenetic tree reconciliation then identifies the combination of events that generates the observed host and parasite phylogenies while minimizing the total costs.

The TreeMap 3.0*β* method considers four of the five events described above (codivergence, duplication, host switching, and parasite loss), and finds the best cost scheme settings while maximizing the probability of codivergence (i.e., minimizing costs assigned to codivergence). It then infers the maximum number of codivergence events and the minimum number of noncodivergence events needed to reconcile the observed host and parasite phylogenies (see Charleston 1998 for the details of the tree‐mapping method). Finally, TreeMap 3.0*β* graphically depicts the differences between host and parasite phylogenies in a “tanglegram” (Page 1994, 1995).

The Jane 4.0 method performs the reconciliation analyses using all five described evolutionary events, whereby the cost of each event is chosen depending on the biological system (see Conow et al. 2010 for the details of the tree‐mapping method). It has been shown that the outcome of event‐based analyses is heavily dependent on the cost scheme employed (Merkle et al. 2010), and choosing a biologically meaningful cost scheme a priori is often difficult (De Vienne et al. 2013). To ensure we would not fail to detect cospeciation because of an inappropriate cost scheme, reconciliation of the host and parasite phylogenies was performed using three different types of cost schemes (see also results Table 1). In the first type, referred to as “equal,” all events were of equal cost. The second type of cost schemes (“codivergence maximization”) maximized the probability for obtaining codivergence by assigning a low cost to codivergence events as suggested by Charleston and Page (2002) and Hendricks et al. (2013). Finally, the third type of cost schemes, called “alternatives,” was used to find scenarios generating good (i.e., low cost) tree reconciliations. In these alternatives, we no longer tried to maximize the probability of codivergence, and instead varied the relative costs associated with codivergence, duplication, and host switch events to obtain evolutionary scenarios with a good fit to the observed data. Other than the cost schemes, we used default settings for all Jane 4.0 parameters as recommended by Conow et al. (2010), with the number of generations (G = 300) set as two times higher than the population size (S = 150). Varying the default settings did not affect our results (data not shown).

**Table 1:** Outcome of cophylogenetic analyses in JANE 4.0, employing different cost schemes

*Model* Biological interpretation	Cost scheme^a^	Analyses^b^	Codivergence	Noncodivergence					Total cost	*P*‐value
			Total number of events	Duplication	Host switch	Parasite loss	Failure to diverge	Total number of events		
*Equal*										
Events of equal costs	11111	2lineages	0	6	17	0	0	23	**23**	1
		Lin1	0	4	8	0	0	12	**12**	1
		Lin2	0	2	8	0	0	10	**10**	1
*Codivergence maximization*										
Codivergence of no cost	01111	2lineages	6	5	12	1	0	18	**18**	0.385
		Lin1	3	3	6	0	0	9	**9**	0.448
		Lin2	2	2	6	0	0	8	**8**	0.678
Codivergence facilitated	−10000	2lineages	9	5	9	9	0	23	−**9**	0.365
		Lin1	4	3	5	7	0	15	−**4**	0.629
		Lin2	4	2	4	4	0	10	−**4**	0.841
Codivergence facilitated	−11111	2lineages	6	5	12	1	0	18	**12**	0.739
		Lin1	3	3	6	0	0	9	**6**	0.996
		Lin2	2	2	6	0	0	8	**6**	0.708
*Alternatives*										
Host switches unlikely	11211	2lineages	1	11	11	0	0	22	**34**	0.115
		Lin1	1	5	6	0	0	11	**18**	0.068
		Lin2	0	6	4	0	0	10	**14**	0.310
No host switches	11N11	2lineages	7	16	NA	36	0	52	**59**	0.133
		Lin1	3	9	NA	27	0	36	**39**	0.610
		Lin2	4	6	NA	8	0	14	**18**	0.100
Maximizing codivergence, minimizing host switches	01211	2lineages	5	8	10	2	0	20	**30**	0.231
		Lin1	3	3	6	0	0	9	**15**	0.211
		Lin2	1	5	4	0	0	9	**13**	0.517
Codivergence and duplication of no cost	00111	2lineages	1	11	11	0	0	22	**11**	0.094
		Lin1	1	5	6	0	0	11	**6**	0.071
		Lin2	0	6	4	0	0	10	**4**	0.319
Duplication of no cost	10111	2lineages	0	11	12	0	0	23	**12**	0.109
		Lin1	0	5	7	0	0	12	**7**	0.022
		Lin2	0	6	4	0	0	10	**4**	0.111

a Costs are ordered as codivergence, duplication, host switch, parasite loss, and failure to diverge.

b For each cost scheme, analyses were performed three times: “2lineages” corresponds to the analyses considering both nematode sublineages together, while “Lin1” and “Lin2” correspond to the analyses considering only one sublineage. Plausible evolutionary scenarios are highlighted in gray.

The values written in bold correspond to the total cost of the various events (including codivergence and noncodivergence events) summed, based of the values indicated in the cost schemes, for each analysis.

Statistical significance of the inferred evolutionary scenarios is evaluated differently in the TreeMap 3.0*β* versus Jane 4.0 methods. To test whether the number of observed codivergence events between hosts and parasites is greater than expected by chance, TreeMap 3.0*β* generates 1000 random parasite trees. The reported *P*‐value then corresponds to the proportion of random parasite trees that result in the same number of, or more, codivergence events than the observed parasite tree (Page 1990, 1994). We also tested whether distances (branch lengths) in associated subtrees of the parasite and the host trees were significantly correlated, as would be expected under codivergence.

In contrast to TreeMap 3.0*β*, Jane 4.0 estimates the observed total cost for the most parsimonious scenario of host–parasite tree reconciliation (under a given cost scheme). The goodness‐of‐fit of this scenario is then evaluated by calculating the total costs for the most parsimonious host–parasite tree reconciliations obtained from each of 1000 randomly generated parasite trees (Conow et al. 2010).

Both TreeMap 3.0*β* and Jane 4.0 use the phylogenies of hosts and their parasites as input. To perform the cophylogenetic analyses implemented in TreeMap 3.0*β*, we used a robust, previously published *Timema* phylogeny (Schwander et al. 2011, 2013), which includes host species for which we did not find any parasites during 9 years of sampling. Because hosts without associated parasites cannot be used in Jane 4.0, we pruned the host phylogeny to comprise only the nine *Timema* species for which we found parasites in analyses with Jane 4.0.

Finally, we also assessed whether geographic distance could contribute to divergence among endoparasites. Pairwise genetic divergences among nematodes were estimated from p‐distances (gaps deleted) in MEGA 6.0 (Tamura et al. 2013). Genetic differentiation due to isolation by distance among endoparasitic nematodes was assessed by conducting Mantel tests in XLSTAT (Addinsoft Version 2015.3.01.19251).

## Results

### Phylogenetic placement of the endoparasitic nematodes

The maximum‐likelihood phylogeny confirmed that the *Timema* endoparasitic nematodes are indeed closely related to species from the family Mermithidae of Nematoda (Clade I; Fig. 2A), and are closely related to the single mermithid ever collected from another stick insect (*Clitarchus*; Yeates and Buckley 2009; Fig. S1). However, identification of nematodes to family levels is difficult, even with DNA evidence. Moreover, the *Timema* nematodes seem to consist of two distinct lineages, although with little bootstrap support (Fig. S1). To take this apparent phylogenetic structure into account, all the following analyses were applied to either the complete set of nematodes (both lineages combined), or by considering the lineages separately.

### Host–parasite cophylogenetic analyses

**Figure 3:**
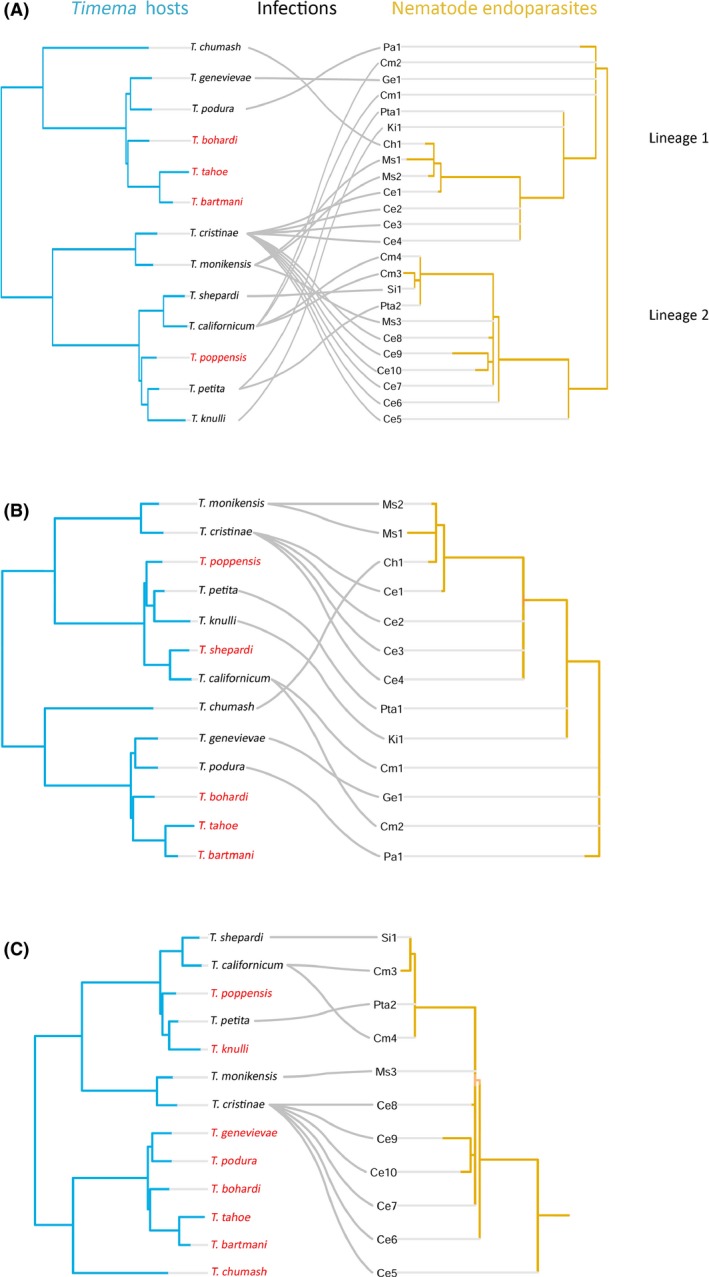
Tanglegrams (generated with TreeMap 3.0*β*) comparing the nematode endoparasite phylogeny (right) to the *Timema* host phylogeny (left) with gray lines indicating host–parasite associations. The two endoparasitic nematode sublineages are combined in (A) and treated separately in (B) and (C).

A visual inspection of the *Timema* host and endoparasite trees does not suggest any coevolution between *Timema* stick insects and their endoparasitic nematodes. This is the case independently of whether the two nematode sublineages are analyzed separately or together (see tanglegrams in Fig. 3). Indeed, neither the method implemented in TreeMap 3.0*β* nor the one implemented in Jane 4.0 provided evidence for coevolution between *Timema* hosts and their parasites. Using TreeMap 3.0*β* for the two nematode sublineages together, we inferred that the most probable coevolutionary history required 16 codivergence events and a minimum of 43 noncodivergence events (23 parasite duplications, nine host switches, and 11 parasite losses). The 16 observed codivergence events were not more frequent than expected by chance (1000 randomizations of the parasite tree, *P*‐value = 0.976). Furthermore, branch lengths in associated subtrees of the parasite and the host tree were not significantly correlated (*P*‐values between 0.22 and 1), in contrast to the pattern expected under codiversification. When considering the two sublineages separately, we detected a maximum of 10 cospeciation events for the first and nine for the second lineage (with respectively 36 and 21 noncodivergence events). These codivergence events were not more frequent than expected by chance (*P*‐value = 0.936 and *P*‐value >0.99).

Similar to the results obtained via the TreeMap 3.0*β* method, we also found no indication of coevolution between hosts and parasites using the methods implemented in Jane 4.0. Analyzing the two nematode sublineages together or separately did not affect the results. All different cost schemes used to infer likely scenarios of host and parasite divergence indicated the absence of a coevolutionary signal (Table 1). Indeed, neither the “equal” cost scheme nor the three “codivergence maximization” cost schemes identified a scenario that would match the observed host and parasite trees better than random trees (*P*‐values between 0.365 and 0.99; Table 1). Plausible evolutionary scenarios with a significantly (or marginally significantly) better match to the observed than to randomized trees were only observed with the “alternative” cost schemes (Table 1). Each of the plausible scenarios inferred either 0 or 1 codivergence events, and 11–22 noncodivergence events (Table 1), indicating, again, the lack of codiversification of endoparasitic nematodes and their *Timema* hosts.

In summary, the lack of a coevolutionary signal in all analyses shows that genetic divergence of the endoparasitic nematodes we collected from *Timema* hosts is not driven by divergence among different host species. Importantly, the lack of a coevolutionary signal between the endoparasites and their hosts is not due to a lack of genetic diversity in the parasites. Indeed, the level of genetic divergence detected among different endoparasites is considerable, with 12% segregating sites and an average sequence divergence of 3.9%.

**Figure 4:**
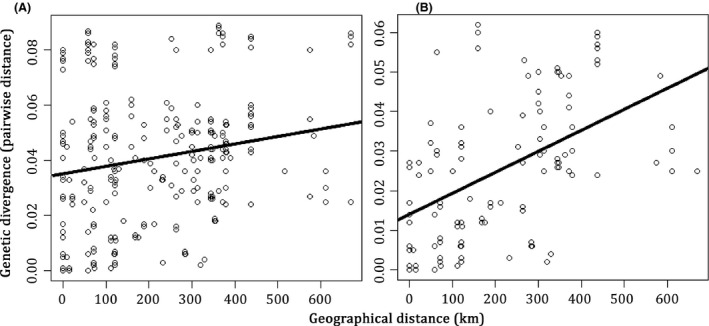
Pairwise genetic distances between endoparasitic nematodes as a function of geographic distances (km) (A) Pairwise distances between sequences from all endoparasitic nematodes (B) Pairwise distances within lineages 1 and 2 (distances between sequences from different lineages are not included).

*Timema* endoparasites appear to diverge because of geographic separation rather than as a consequence of host‐driven divergence. Irrespective of the identity of the host, we observed strong isolation by distance between the endoparasitic nematodes (Mantel test with 10,000 permutations: *r* = 0.13, *P*‐value <0.0001; Fig. 4A). The pattern was even stronger when both nematode sublineages were analyzed separately (partial Mantel test with 10,000 permutations: *r* = 0.24, *P*‐value <0.0001; Fig. 4B). Indeed, we found genetically similar nematodes parasitizing very distinct *Timema* species (Fig. 3), as nicely illustrated by genetically similar parasites infecting the phylogenetically distinct hosts *T. chumash* and *T. monikensis* at a location where the two hosts co‐occur.

## Discussion

Coevolution, the process of reciprocal adaptation between ecologically interacting species, is considered as a key force generating biological diversity (e.g., Clarke 1976; Price 1980; Kiester et al. 1984; Buckling and Rainey 2002; Thompson et al. 2005; Ricklefs 2010; Yoder and Nuismer 2010; Masri et al. 2015). In this study we identified a new group of endoparasitic nematodes, infecting at least nine species of *Timema* stick insects throughout California, as relatives of mermithid nematodes. This is only the second report of mermithid (or mermithid‐like) nematodes infecting stick insects, after Yeates and Buckley (2009) found a mermithid nematode infecting a *Clitarchus* stick insect in New Zealand. We found that this mermithid is closely related to *Timema* endoparasites, suggesting few or perhaps even only a single colonization of phasmatodean hosts by mermithids.

In natural *Timema* populations, nematodes emerged from typically <1.2% of the host individuals. Obviously, these low emergence rates only include cases where the parasites have managed to infect the *Timema* hosts and successfully completed their development. They do not take into account the cases where hosts died prior to parasite emergence, or cases where infected hosts managed to suppress parasite development.

The phylogenetic analyses of the endoparasitic nematodes suggested the presence of two sublineages. Independently of whether these sublineages were considered separately or jointly, and independently of the cophylogenetic analyses conducted (TreeMap 3.0*β* and Jane 4.0 with a broad range of cost settings), we found a complete lack of codivergence between the parasites and their *Timema* hosts. We conducted over 30 cophylogenetic analyses, but the level of congruence between the host and parasite phylogenies was never higher than expected by chance.

It is very unlikely that the absence of host–parasite codiversification is due to incorrect phylogenies of either the host or the parasite. The *Timema* host phylogeny is very robust (Schwander et al. 2011, 2013). For the parasite phylogeny, although several nodes are weakly supported, topology errors for the weakly supported nodes would not influence the main result. Indeed, there were many noncodivergence events (Table 1) that concern the well supported nodes in the parasite tree (e.g., nematodes infecting *T. cristinae* hosts*,* in Fig. 3A) such that minor topology changes at poorly supported nodes would not change the main conclusion of little or no host–parasite codiversification.

Similarly, the lack of host–parasite codiversification is not due to little genetic divergence within the hosts or parasites. Nine different *Timema* species (some of which have diverged for over 20 million years; Sandoval et al. 1998) from a large geographic area (Fig. 1; the two most distant sampling points are separated by 670 km) are infected by these endoparasites. The genetic variation among nematodes is also substantial (average pairwise sequence divergence of 3.9%). Furthermore, we found significant isolation by distance among *Timema* nematodes (Fig. 4). Hence, nematode genetic divergence seems driven much more by geographic separation than by coevolution and adaptation to their hosts, indicating the absence of “ecological speciation” in this system.

The review of a number of host–parasite systems by Barker (1994) suggested that codiversification of parasites with their hosts seems to mainly happen when the hosts are allopatric. This would be the case for *Timema* as there is overall little overlap in the distribution ranges of different *Timema* species (Law and Crespi 2002). But despite these apparently favorable environmental conditions, we did not find the expected codiversification.

Similar to the lack of codiversification between *Timema* hosts and their endoparasitic nematodes, other parasite species known to be strongly host‐specific also diverged independently of their host. For example, flatworms in the genus *Lamellodiscus* infect different fish species in *Sparidae* family, with no apparent phylogenetic congruence between the parasites and their hosts (Desdevises et al. 2002). The same observation was made on fish parasitic copepods (Paterson and Poulin 1999) and trematodes (Cribb et al. 2001) or monogenea platyhelminthes (Huyse and Volckaert 2005). In each of these systems, the lack of codiversification was suggested to be due to the ecology of the parasites, with short periods outside the hosts, as well as the aquatic environment, which would greatly facilitate parasite dispersal and thus potentially host switches. However, such frequent host switches would be less likely in terrestrial systems like *Timema*. Furthermore, *Timema* are wingless and do not disperse over long distances (Sandoval 1994; Schwander et al. 2010). As mentioned above, different *Timema* species also feature quite distinct distribution ranges, further constraining the opportunity for host‐mediated parasite dispersal and exposure of parasites to alternative hosts species. A notable exception to this general pattern stems from the two distantly related species *T. chumash* and *T. monikensis,* which share a similar nematode parasite strain in the location where these two species co‐occur (Fig. 1A).

In addition to frequent host switches, several other ecological factors may also contribute to the noncongruence of host and parasite trees. (Clayton et al. 2004; Whiteman et al. 2007; Hoberg and Brooks 2008; Nieberding et al. 2010) for instance, a number of studies highlighted the fact that macroparasites often feature higher mutation rates, smaller effective population sizes, and limited dispersal abilities relative to their hosts (e.g., McDonald and Linde 2002; Criscione and Blouin 2004; 2005) Poulin 2011). The implications are that genetic drift can be very pronounced in parasites and generate extensive spatial genetic structure independently of divergence among parasite strains infecting different hosts. Drift might indeed be an important mechanism constraining codivergence of *Timema* endoparasitic nematodes and their hosts. The endoparasitic life cycle, as well as the apparently low frequency of infections in natural stick insect populations (<1.2%), suggest that the endoparasites’ population sizes might be orders of magnitude smaller than their hosts’ – unless the same endoparasites also infect non‐*Timema* hosts.

A broad host range including species from other genera or even other insect orders could also explain the lack of codiversification between the endoparasites and *Timema*. Although the ecology and biology of the *Timema* endoparasites have never been studied specifically, the ecology of a range of mermithid nematode species has been well documented (e.g., Nickle 1972; Poinar 1975; Poinar et al. 1976; Platzer 2007). Mermithid species are typically characterized by strong host specificity (Stoffolano 1973; Kennedy 1975; Rohde 1979, 2002; Noble et al. 1989; Sasal et al. 1998) while the family as a whole is cosmopolitan and infects a broad range of invertebrates (Poinar 1985; Mebrahtu 1987; Kaiser 1991; Vandergast and Roderick 2003; Nikdel et al. 2011; Gradinarov 2014). Nevertheless, it remains possible that some mermithid species are generalists and use a broad range of hosts. A mixture of highly host‐specific and generalist species is, for example, known in parasitoid wasps, which, similar to mermithid nematodes, kill their hosts at emergence, preventing reproduction of their hosts (see Eggleton and Gaston 1990 and Godfray 1994 for a discussion of further similarities between parasitoid wasps and parasitic nematodes). Future studies on the ecology of the *Timema* endoparasitic nematodes may shed light on these questions.

Thus far, the vast majority of examples revealing strong codiversification between parasites and their hosts stem from pocket gophers and their chewing lice (e.g., Hafner and Nadler 1988; Hafner and Page 1995; Demastes et al. 2002; Hafner et al. 2003) and from swiftlets and their parasitic lice (Page et al. 1998). In both cases, the close relationship between the hosts and their parasites led to identical topologies of the phylogenies, indicating that the hosts and parasites speciated in perfect synchrony (a pattern known as the Fahrenholz's rule). However, given the accumulating evidence from other host–parasite systems (e.g., see review by De Vienne et al., 2013), including *Timema* and their nematode endoparasites, the pocket gophers/swiftlets–lice systems seem to represent a fairly unusual pattern. Therefore, explaining the frequent lack of codiversification between parasites and their hosts at macroevolutionary scales, even though there is a large body of evidence for coevolution between hosts and parasites within populations (microevolutionary scale, e.g., Brooks 1979; Anderson and May 1982; Kaltz and Shykoff 1998; Decaestecker et al. 2007), remains a challenge for future studies. Indeed, as previously suggested by De Vienne et al. (2013), codiversification with hosts does not seem to be the predominant mode of speciation in parasites, despite the well‐documented occurrence of reciprocal selection over short timescales. There is thus a crucial need for studies linking micro‐ versus macroevolutionary dynamics in host–parasite interactions.

In conclusion, this study reports a new group of endoparasitic nematodes, related to the mermithid family, infecting several species of *Timema* stick insects. We found no codiversification between these parasites and their hosts, even though codiversification might be expected given the close interaction between the parasites and their hosts and the dramatic fitness costs of infection. Instead, geographic distance seems to play a more important role than host‐related adaptations in driving genetic differentiation between parasites in this system.

## Conflict of Interest

None declared.

## Supporting information


**Table S1.** Samples iD, host species and sequence information of the nematodes used in this study.

**Figure S1.** Maximum likelihood phylogeny of 48 Mermithid nematodes from Clade I.
