# Supplementary material for: Nematode endoparasites do not codiversify with their stick insect hosts
Source: Ecol Evol. 2016 Jul 10;6(15):5446–58. doi: 10.1002/ece3.2264 (PMC4984516; doi:10.1002/ece3.2264)
Supplement: Supplementary file 1 — Table S1. Samples iD, host species and sequence information of the nematodes used in this study. [file ECE3-6-5446-s001.docx]

Supplementary materials

**Table S1. Samples iD, host species and sequence information of the nematodes used in this study**

| Sample iD | Source | Clade (from Blaxter et al., 1998) | Dataset/  Figure | GenBank Accession numbers |
| --- | --- | --- | --- | --- |
| *Anatonchus tridentatus* | GenBank | I | 2, S1 | AJ966474 |
| *Bathyodontus cylindricus* | GenBank | I | 2, S1 | AY552964 |
| *Bathyodontus mirus* | GenBank | I | 2, S1 | AY284744 |
| *Clarkus papillatus* | GenBank | I | 2, S1 | AY284748 |
| *Granonchulus* sp. | GenBank | I | 2, S1 | AY593953 |
| *Isomermis lairdi* | GenBank | I | 2, S1 | FN400900 |
| *Longidorus elongates* | GenBank | I | 2, S1 | AY687992 |
| *Longidorus grandis* | GenBank | I | 2, S1 | AY283165 |
| *Mermis nigrescens* | GenBank | I | 2, S1 | AF036641 |
| *Mermis* sp. | GenBank | I | 2, S1 | FJ973464 |
| *Mermithid* sp. | GenBank | I | 2, S1 | AY284743 |
| *Mermithidae* | GenBank | I | 2, S1 | FJ040480 |
| *Mononchus truncates* | GenBank | I | 2, S1 | AJ966493 |
| *Mononchus tunbridgensis* | GenBank | I | 2, S1 | AY593954 |
| *Mylonchulus* sp. | GenBank | I | 2, S1 | AY284761 |
| *Mylonchulus arenicolus* | GenBank | I | 2, S1 | AF036596 |
| *Paralongidorus maximus* | GenBank | I | 2, S1 | AJ875152 |
| *Soboliphyme baturini* | GenBank | I | 2, S1 | AY277895 |
| *Trichuris muris* | GenBank | I | 2, S1 | AF036637 |
| *Xiphinema bakeri* | GenBank | I | 2, S1 | AY283173 |
| *Xiphinema krugi* | GenBank | I | 2, S1 | AY297828 |
| *Xiphinema rivesi* | GenBank | I | 2, S1 | AM086673 |
| *Xiphinema taylori* | GenBank | I | 2, S1 | AM086676 |
| *Xiphinema simile* | GenBank | I | 2, S1 | AM086681 |
| *Dirofilaria immitis* | GenBank | III | 2 | AF036638 |
| *Raillietnema* sp. | GenBank | III | 2 | DQ503461 |
| *Wellcomia slamensis* | GenBank | III | 2 | EF180079 |
| *Acrobeles complexus* | GenBank | IV | 2 | AY284671 |
| *Bursaphelenchus mucronatus* | GenBank | IV | 2 | AY508022 |
| *Strongyloides ratti* | GenBank | IV | 2 | SRU81581 |
| *Cephaloboides* sp. | GenBank | V | 2 | AF083027 |
| *Rhabditis colombiana* | GenBank | V | 2 | AY751546 |
| *Syngamus trachea* | GenBank | V | 2 | AJ920344 |
| Ce1 | Current study | I | 1, 2, 3, 4, S1 | KX301041 |
| Ce2 | Current study | I | 1, 2, 3, 4, S1 | KX301053 |
| Ce3 | Current study | I | 1, 2, 3, 4, S1 | KX301054 |
| Ce4 | Current study | I | 1, 2, 3, 4, S1 | KX301055 |
| Ce5 | Current study | I | 1, 2, 3, 4, S1 | KX301043 |
| Ce6 | Current study | I | 1, 2, 3, 4, S1 | KX301051 |
| Ce7 | Current study | I | 1, 2, 3, 4, S1 | KX301042 |
| Ce8 | Current study | I | 1, 2, 3, 4, S1 | KX301052 |
| Ce9 | Current study | I | 1, 2, 3, 4, S1 | KX301046 |
| Ce10 | Current study | I | 1, 2, 3, 4, S1 | KX301050 |
| Ms1 | Current study | I | 1, 2, 3, 4, S1 | KX301039 |
| Ms2 | Current study | I | 1, 2, 3, 4, S1 | KX301040 |
| Ms3 | Current study | I | 1, 2, 3, 4, S1 | KX301044 |
| Cm1 | Current study | I | 1, 2, 3, 4, S1 | KX301047 |
| Cm2 | Current study | I | 1, 2, 3, 4, S1 | KX301045 |
| Cm3 | Current study | I | 1, 2, 3, 4, S1 | KX301049 |
| Cm4 | Current study | I | 1, 2, 3, 4, S1 | KX301048 |
| Si1 | Current study | I | 1, 2, 3, 4, S1 | KX301056 |
| Pa1 | Current study | I | 1, 2, 3, 4, S1 | KX301060 |
| Ge1 | Current study | I | 1, 2, 3, 4, S1 | KX301061 |
| Ch1 | Current study | I | 1, 2, 3, 4, S1 | KX301038 |
| Ki1 | Current study | I | 1, 2, 3, 4, S1 | KX301059 |
| Pta1 | Current study | I | 1, 2, 3, 4, S1 | KX301058 |
| Pta2 | Current study | I | 1, 2, 3, 4, S1 | KX301057 |


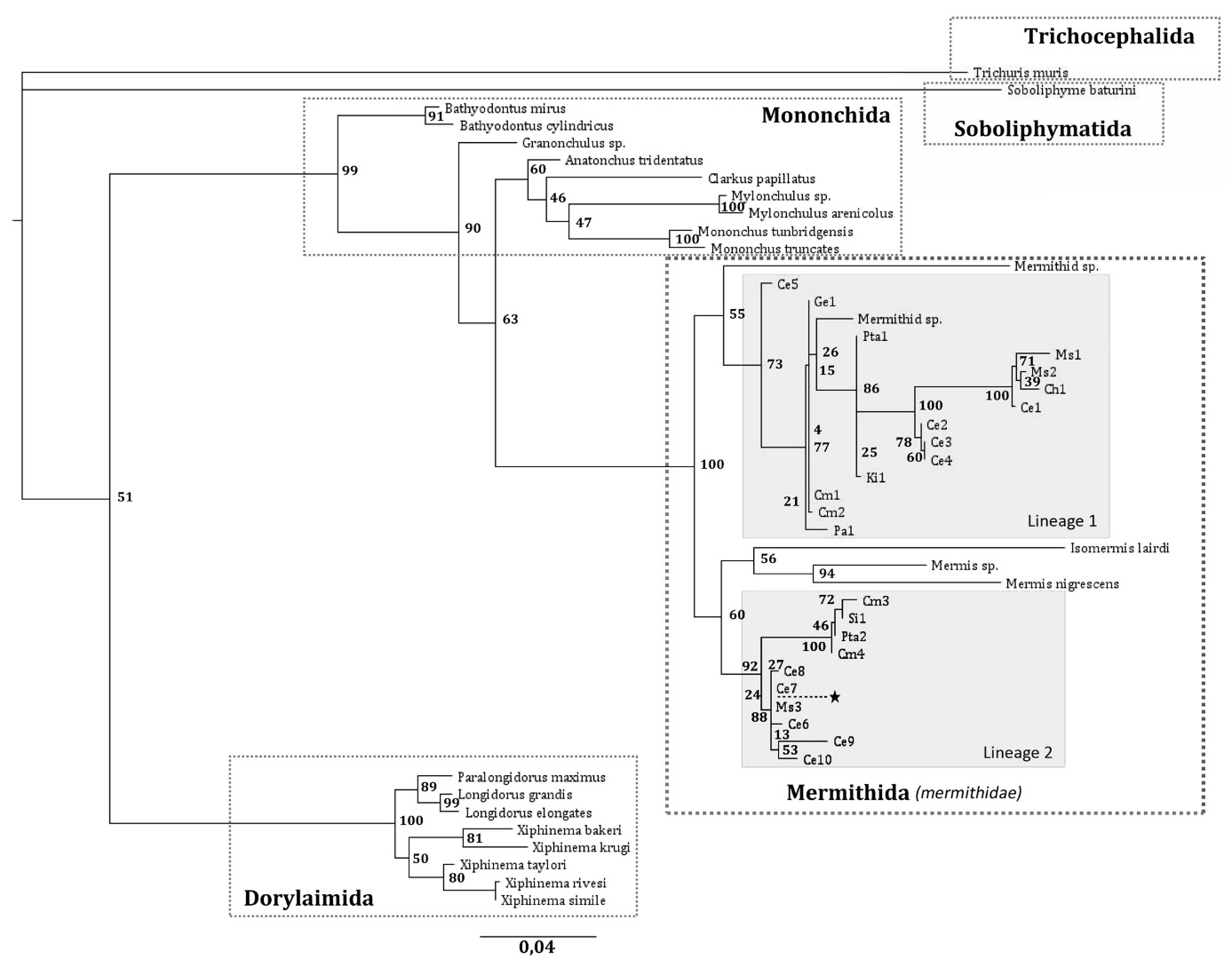


**Figure S1.** **Maximum likelihood phylogeny of 48 Mermithid nematodes from Clade I.** Among the 48 sequences, 24 are from the endoparasitic nematodes using *Timema* stick insects as hosts (sequences highlited in grey), and 24 are from previously published sequences (Ross *et al*., 2010). The different orders of Clade I are in bold and delineated by dotted lines. Bootstrap support was calculated using 1000 replicates. The black star indicates the position of the endoparasitic mermithid collected from a *Clitarchus sp* stick insect by Yeates and Buckley (2009).
