# Supplementary figures and images for: Nematode endoparasites do not codiversify with their stick insect hosts
Source: Ecol Evol. 2016 Jul 10;6(15):5446–58. doi: 10.1002/ece3.2264 (PMC4984516; doi:10.1002/ece3.2264)

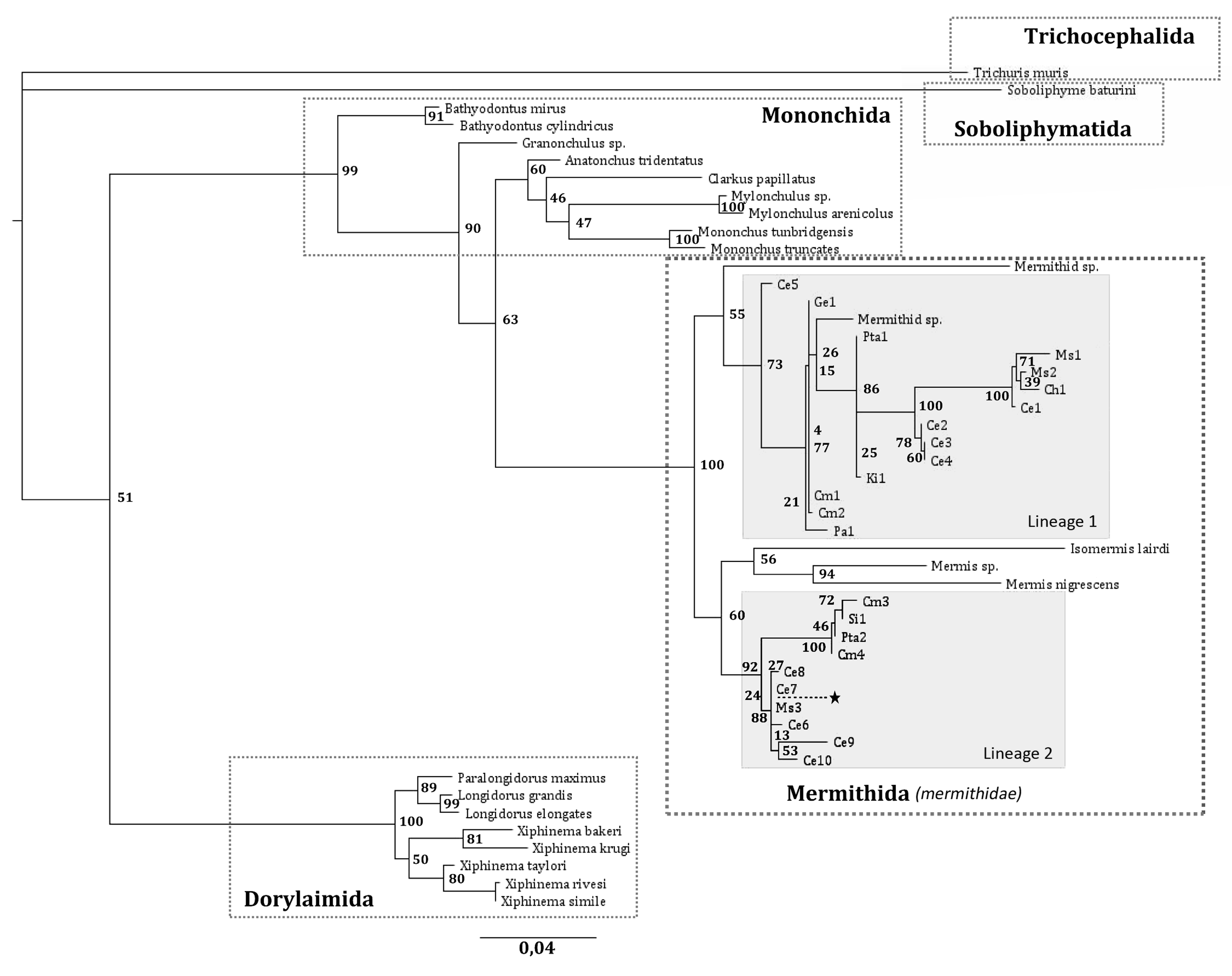

Supplement: Supplementary file 2 — Figure S1. Maximum likelihood phylogeny of 48 Mermithid nematodes from Clade I. [file ECE3-6-5446-s002.jpg]
